# Influence of physical exercise on health improvement in older adult cancer patients: a systematic review

**DOI:** 10.3389/fpubh.2025.1525021

**Published:** 2025-08-11

**Authors:** Rafael Corrêa, Cristina Vaz de Almeida, Vasco da Fonseca, Benjamin Miranda Tabak

**Affiliations:** ^1^School of Public Policy and Government, Getulio Vargas Foundation, Brasília, Brazil; ^2^Center for Administration and Public Policy and Institute of Social and Political Sciences, University of Lisbon, Lisbon, Portugal; ^3^Institute of Social and Political Sciences, University of Lisbon, Lisbon, Portugal

**Keywords:** cancer, quality of life, polypharmacy, health literacy, older adult

## Abstract

**Introduction:**

Physical exercise has shown evidence in the recovery of cancer treatment. Therefore, interventions that influence these patients to adhere to physical exercise may prove beneficial. This study aimed to identify the influence of physical exercise on health improvement in older adult cancer patients.

**Methods:**

The inclusion criteria are according to the PICOS strategy. We conducted this systematic review by searching the electronic databases PubMed, Web of Science, Scopus, and Cochrane on February 21, 2024. The systematic review was registered in PROSPERO (CRD42024508547). Each article included in the review was assessed for study quality using the Joanna Briggs Institute (JBI) Critical Appraisal Tools. The data was presented in tables and described qualitatively in the text.

**Results:**

The systematic search in the databases identified 3,593 articles. We filtered these articles and obtained 15 studies focused on our research question. Exercise interventions show evidence that individual, group, and home exercise, as well as multimodal exercise programs, have positive outcomes for the physical and mental health and quality of life (QL) of older adult cancer patients. However, the studies had limitations regarding polypharmacy and health literacy measures.

**Discussion:**

Physical exercise interventions showed positive results in the recovery of physical and mental health and the quality of life of older adult cancer patients. Future studies could explore physical activity interventions with polypharmacy and health literacy measurement tools to identify specific interventions that optimize the health and quality of life of older adult cancer patients.

**Systematic review registration:**

PROSPERO (CRD42024508547), https://www.crd.york.ac.uk/PROSPERO/view/CRD42024508547.

## 1 Introduction

In this paper, we conduct a systematic review focusing on interventions that employ physical exercise as part of treating older adult cancer patients. The lack of adequate literature on the effects of these exercise programs on health of older adult cancer patients motivates the choice of this review (1, 2). We focus on recent literature (last 5 years) as we can focus on the recent findings and how they may help treat older adult cancer patients.

Healthy habits such as exercising may be related to the development of a variety of diseases (2). One example is that most cancer diagnoses are identified in older adult adults together with other health comorbidities related to the cancer condition, which are the result of their unhealthy habits (1, 2). The process of being diagnosed with cancer influences the likelihood of developing mood and anxiety disorders, as well as physical and emotional disturbances, influencing wellbeing and quality of life (QL) (3, 4). This distress lowers the likelihood of adhering to healthy habits such as exercise (4).

One of the most frequent symptoms in older adult cancer patients is the decrease in muscle mass and, consequently, the negative impact on functional capacity, conditions increased by factors such as sedentary lifestyles, unhealthy habits, and growing age itself (5). All these factors are considered to be predictors of complications in disease progression, postoperative complications, and increased morbidity in cancer patients (6, 7). Physical exercises would reduce these effects and help improve the quality of life of these patients (6).

Another point to highlight about concerns about the development of cancer in the older adult is the risk of comorbidity due to the weakening of the immune system (8). It is estimated that the number of older adult cancer patients increases every year, thus increasing complications from other diseases and, consequently, cancer mortality rates (2, 9, 10). This trend suggests that research on how a variety of interventions may help improve these numbers is essential.

The identification of the above conditions and those of the increased toxicity rates from conventional treatment based on chemotherapy or radiotherapy and pharmacological interventions, increase in the adverse effects in the cancer treatment process in older adult patients (11). Research in health sciences has highlighted the importance of physical exercise and behavioral sciences in improving the current state of the disease, taking into account its physical, emotional, and social aspects (12, 13), thus contributing to the development of a cancer treatment process that encompasses the patient’s quality of life (3).

Research carried out with cancer patients to identify the effects of exercise programs in promoting physical rehabilitation has shown positive evidence (14–16).

Studies have recommended physical exercise for cancer patients to prevent the worsening of the disease, with moderate aerobic exercise for 150 min a week and/or vigorous exercise for 75 min a week in parallel with resistance exercise twice a week being recommended, but for older adult cancer patients this goal is difficult to achieve (14, 17). However, there are still discussions about the benefits of exercise modalities for cancer patients (18, 19).

In addition to the benefits on the side effects of conventional cancer treatment, aerobic and resistance exercise has been shown to improve muscle strength and functional performance (20, 21), as well as improving inflammatory conditions (18, 22), in older adult cancer patients and other chronic diseases in a systematic review study. However, continued adherence to exercise is still a concern (23, 24).

Another modality that has stood out in studies of intervention for older adult cancer patients is the multimodal, which consists of combining physical exercise with strategies to reduce stress and anxiety, nutritional guidance, and monitoring to improve rehabilitation and the general functioning of patients (6, 25).

Physical exercise programs have also shown evidence of the positive effects of treatment, such as reduced anxiety (3, 26), depression (23, 27), reduced stress (28), self-esteem (29, 30), increasing patient survival time (23, 31) and patients’ quality of life (9, 32). Many of the studies were carried out with middle-aged adults, and there is a gap in the literature when it comes to conducting experiments with older adult cancer patients (3, 9, 33).

Therefore, practicing physical exercise and maintaining healthy habits is important in the treatment process of older adult cancer patients (34, 35). Although it is still a challenge for patients (36). Therefore, it is important to analyze the evidence produced by physical exercise intervention programs and their benefits in improving the health and QL of older adult cancer patients. Our paper contributes to filling this gap by focusing on the effects of physical exercise on older adult cancer patients. These patients are much harder to adhere to such interventions due to a variety of factors, and understanding the current state of interventions is essential for the development of public health policies focusing on a segment of the population that is becoming increasingly numerous (36).

## 2 Materials and methods

A systematic review, following the Preferred Reporting Items for Systematic Reviews and Meta-Analyses (PRISMA) standardized reporting guidelines (37) (Supplementary Table S1), was conducted to explore the influence of physical exercise in older adult cancer patients. The systematic review was registered in PROSPERO (CRD42024508547). The review question is: “What influence do intervention studies on physical exercise have on improving the health (physical and mental) and QL of older adult cancer patients?”

### 2.1 Search strategy

Extensive and systematic searches were conducted across 4 databases—Web of Science, Cochrane, Scopus, and PubMed—on 21st February 2024. The strategy was created under five keywords: Older adult, Cancer, Exercise, Health literacy, and Polypharmacy. The final combination of key search terms and medical subject headings (MESH) is used depending on the searched database. The entire search strategy for each database has been provided in Table 1.

**Table 1:** Search strategy in each electronic database.

Database	Studies influence of intervention on chronic diseases
MEDLINE/PUBMED	(((Polypharmacy) OR (Health literacy)) OR (Exerc*)) AND (Cancer) Filters: Full text, Clinical Trial, Controlled Clinical Trial, Randomized Controlled Trial, in the last 5 years, English, Aged: 60+ years, 80 and over: 80+ years.
SCOPUS	(((Polypharmacy) OR (Health literacy)) OR (Exerc*)) AND (Cancer) Filters: Full text, Clinical Trial, Controlled Clinical Trial, Randomized Controlled Trial, in the last 5 years, English, Aged: 60+ years, 80 and over: 80+ years.
Web of Science	Polypharmacy (All Fields) OR Health literacy (All Fields) OR Exerc* (All Fields) AND Cancer (All Fields) and Randomized Con- trolled Trial (Search within the topic) and Child* (Exclude – Search within the topic) and Teenage* (Exclude – Search within the topic) and Young* (Exclude – Search within the topic) and Adolesc* (Exclude – Search within the topic) and Adult* (Exclude – Search within the topic) and Article (Document Types) and 2024 or 2023 or 2022 or 2021 or 2020 (Publication Years).
Cochrane	Polypharmacy in Title Abstract Keyword OR Health literacy in Title Abstract Keyword OR Exerc* in Title Abstract Keyword AND Cancer in Title Abstract Keyword AND elder* in Title Ab- stract Keyword - with Publication Year from 2020 to 2024, with Cochrane Library publication date Between Jan 2020 and Jan 2024, in Trials (Word variations have been searched)

### 2.2 Eligibility criteria

The study was based on the PICOS strategy, which included articles from intervention studies and randomized controlled trials (RCTs) published in full in English from January 2020 to February 2024, considering the most recent interventions in physical exercise in older adult cancer patients.

According to the PICOS strategy, the inclusion criteria were: (1) Population: The studies should include individuals aged above 60 and diagnosed with cancer. (2) Intervention: interventions using physical exercise. (3) Control/comparison: health literacy, polypharmacy measures. (4) Outcome: improved health and quality of life, and (5) Study design: RCT studies.

### 2.3 Study selection

All the articles retrieved through the database search were imported into the Rayyan software, where duplicates were removed. Two reviewers examined the titles and abstracts, and the same reviewers examined the full-text articles. A third reviewer was consulted to resolve any discrepancies.

### 2.4 Data extraction

Data extraction similarly took place, following the independent analysis process between authors and a third author’s supervision. The data extracted were author/year/country, study design and sample, instruments, intervention, study quality, and results.

### 2.5 Quality assessment

Each article included in the review was assessed for study quality using the Joanna Briggs Institute (JBI) Critical Appraisal Tools (38). The tool was chosen due to the heterogeneity of the interventions and results of the included studies. The JBI was mainly used to assess the risk of bias. Two authors performed the quality assessment scoring again independently and supervised by a third author when disagreements occurred. The instrument contains 13 items that assess internal validity, biases related to participant retention, and biases related to participant retention. The questions have a Likert scale of 0–1 where the answer “yes” corresponds to 1, the answers “no” and “unclear” correspond to 0, and the answer N/A is not counted when calculating the final percentage. The final percentage is calculated from the sum of the raw items for each question divided by the number of total items multiplied by 100 (38).

Methodological quality was calculated using the following classification: a score of over 80% was classified as high quality (or low bias), 60–79% as moderate quality (moderate bias), and 30–59% as poor quality (high risk of bias) (38). The data is presented in table format and described qualitatively through discussion in the text.

## 3 Results

**Figure 1:**
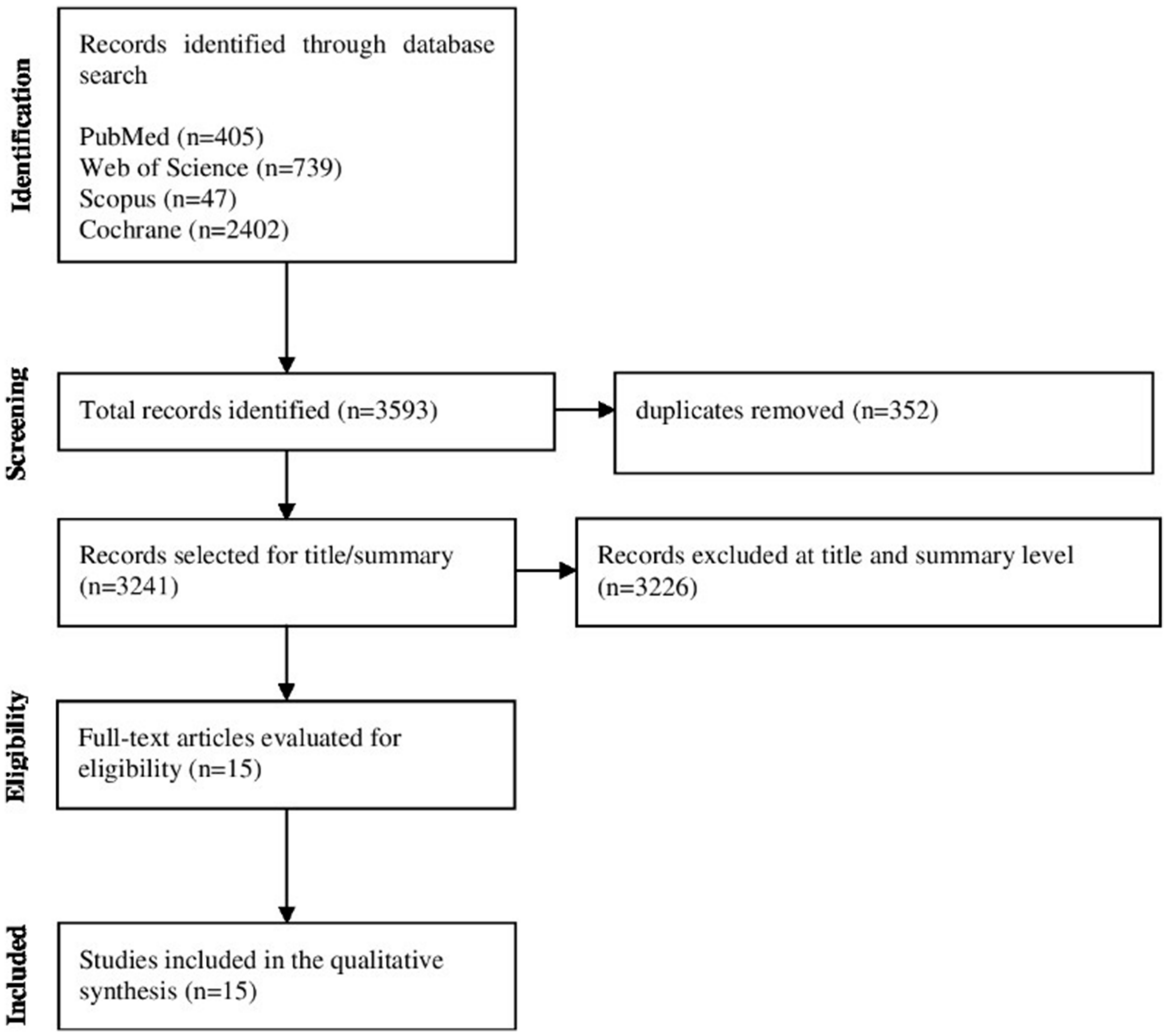
Study flowchart.

3,593 articles were identified using the search strategies in the electronic databases, and the articles were exported to Rayyan, removing duplicates (*n* = 352). The titles, followed by the abstracts, were analyzed according to the inclusion criteria and the PICOS strategy, and 3,226 articles were excluded. Due to the use of a broad search strategy in the databases, the use of exclusion criteria of RCT studies, and the publication period within the last 5 years, the number of exclusions was significant at this screening stage. The final sample consisted of 15 articles that were selected for full reading. Figure 1 shows the flowchart of the article screening and selection process.

Of the 15 studies included in this review, six originated from the USA (3, 14, 20, 39–41), three from Denmark (9, 23, 42), two from Canada (6, 43), one from Spain (29), one from Belgium (18), one from Poland (44) and one from the Netherlands (34). Studies were published between 2019 and 2022 (3, 6, 9, 14, 18, 20, 23, 29, 34, 39–44).

The participants had various cancer diagnoses: six studies had lung cancer (6, 9, 14, 39, 42, 44), three had prostate cancer (18, 20, 23), three had breast cancer (29, 40, 41), one had intra-abdominal and thoracic cancer (43), one had leukemia (3) and one had gastrointestinal, female organ, and urogenital organ malignancies (34). The sample sizes in the studies ranged from 26 participants (20, 44) to 1.326 participants (34).

The average age of the study participants in the sample was 66 years, with age >60 as the study inclusion criterion. As for the gender of the participants, the majority were women (3, 9, 39, 42, 43), followed by three studies which had a larger sample of men (6, 34, 44), four studies only with men (14, 18, 20, 23) and three studies were carried out only with women (29, 40, 41). The sample sizes of the studies varied between 26 and 661 participants.

Physical exercise interventions for older adult cancer patients were identified as individual exercise (14, 18, 20, 44), group exercise (23, 29), home-based exercise (3, 43) and multimodal program of exercise (6, 9, 34, 39–42).

The individual exercise intervention presented strategies such as aerobic and resistance training prescription with supervision (14, 20), high-intensity interval training, and resistance training (18), physical exercise, and running cycles (44), with results in an improvement in cardiorespiratory fitness (CRF), leg strength, and functional capacity (20), declines in peak oxygen consumption (VO2peak), muscle strength, fatigue, and quality of life, and improvement in the depression assessment (14), increase in cancer-treatment-related fatigue (CTRF) and functional capacity (18), more significant deterioration in physical wellbeing and quality of life in the control group (44).

Group exercise developed interventions with strategies in strength training, aqua aerobics, aerobic exercise (29), football group (23), demonstrating evidence of improvement in the health survey (SF-12), physical functioning and limitations, pain relief, emotional and mental dimensions, general health, vitality, and social functioning, and decrease in vitality and mental health (29), improvement in total hip bone mineral density (BMD) and shorter hospital stays (23).

The home exercise intervention included strategies such as a prehabilitation program with remote support and nutritional guidance, which did not show improvements in recovery after cancer surgery in frail older adult people (43). However, the intervention in the home program of progressive walking and low-to-moderate intensity resistance training showed improvements in anxiety scores (STAI), mood state (POMS), social and emotional wellbeing (3).

Finally, the multimodal strategy of the exercise program featured methodologies such as the early and late rehabilitation group with exercise sessions, individual counseling sessions, and group classes on health-promoting behaviors (42), teaching session, a FitBit®, individual walking goals; and gain-framing text messages (39), group exercise, protein supplementation, a home walking program, and support and advice (9), Group-based exercise intervention, SG + Fitbit with post-intervention semi-structured interviews (40, 41), showing results in an improvement in quality of life (42), increased Physical Activity (PA), the quality of life function domain, dyspnea and biomarkers for lung cancer biology, improvement in soluble Programmed Cell death Protein-1 (sPD-1) in blood samples (39), improvement in the chair stand test, physical resistance, handgrip strength, physical activity, symptom burden, symptoms of depression and anxiety, global health status, quality of life, and lean body mass (9), identification of factors involved in the process of recruitment, participation, and acceptability of program participants (40) and improved the physical performance of Breast Cancer Survivors (BCSs), improved in the walk, African American (AA) exercise (41) (Table 2). See more information in Supplementary Table S2 about the main results of included studies.

**Table 2:** A summary of the articles is included in the final sample.

Type of intervention in physical exercise	Studies	Influence on health improvement in older adults with cancer
Individual exercise	Harman et al., 2021 (USA) (14)	Improvements in declines in maximum oxygen consumption, muscle strength, fatigue, and quality of life.
	Piraux et al., 2020 (Belgium) (18)	Increased fatigue related to cancer treatment and functional capacity.
	Harrison et al., 2022 (USA) (20)	It improved cardiorespiratory fitness, leg strength, and functional capacity.
	Rutkowska et al., 2021 (Poland) (44)	Less deterioration in physical well- being and quality of life.
Group exercise	Bjerre et al., 2019 (Denmark) (23)	Improved total hip bone mineral density, shorter hospital stays, and better mental health and weight loss.
	García-Soidán et al., 2020 (Spain) (29)	Improved physical functioning and limitations, pain relief, emotional and mental dimensions, general health, vitality, and social functioning.
Home-based exercise	Loh et al., 2019 (USA) (3)	Improvements in anxiety, mood, social wellbeing, and emotional wellbeing.
	McIsaac et al., 2022 (Canada) (43)	It showed no improvement in recovery after cancer surgery in frail older adults.
Multimodal program of exercise	Ferreira et al., 2021 (Canada) (6)	Increased adherence to home exercises, nutritional supplements, and preoperative assessment.
	Mikkelsen et al., 2022 (Denmark) (9)	Improved physical endurance, handgrip strength, physical activity, symptom burden, depression and anxiety symptoms, overall health status, and lean body mass.
	IJsbrandy et al., 2022 (Netherlands) (34)	Improved screening with the Distress Thermometer.
	Bade et al., 2021 (USA) (39)	Increased physical activity, quality of life, dyspnea, and biomarkers for lung cancer biology.
	Owusu et al., 2020 (USA) (40)	Identify the factors involved in the recruitment process and the participation and acceptability of program participants.
	Owusu et al., 2022 (USA) (41)	It improved physical performance.
	Sommer et al., 2020 (Denmark) (42)	Improved quality of life.

The average score for the methodological quality of the studies was 80%. Eight studies were rated as high-quality (9, 18, 20, 23, 41, 43, 44), six studies as moderate (3, 29, 34, 39, 40, 42) and one study was poor (14). See Table 3 for the quality ratings of the studies.

**Table 3:** Analysis of the domains of the JBI assessment checklist for the included studies.

Internal validity bias related to:														
Studies	Selection and allocation			Administration of intervention			Assessment, detection and measurement of the outcomes			Participant retention	Statistical conclusion validity			Overall validity of the study (%)
	1	2	3	4	5	6	7	8	9	10	11	12	13	
Bade et al., 2021 (USA) (39)	Y	N	Y	N	N	Y	N	Y	Y	Y	Y	Y	Y	69
Bjerre et al., 2019 (Denmark) (23)	Y	Y	Y	Y	Y	Y	Y	Y	Y	Y	Y	Y	Y	100
Ferreira et al., 2021 (Canada) (6)	Y	Y	Y	Y	Y	Y	Y	Y	Y	Y	Y	Y	Y	100
García-Soidán et al., 2020 (Spain) (29)	Y	Y	Y	N	N	Y	Y	Y	Y	N	Y	Y	Y	77
Harman et al., 2021 (USA) (14)	N	N	Y	N	N	Y	N	Y	Y	N	N	Y	N	38
Harrison et al., 2022 (USA) (20)	Y	Y	Y	Y	Y	Y	N	Y	Y	N	Y	Y	Y	85
IJsbrandy et al., 2022 (Netherlands) (34)	Y	Y	Y	N	N	Y	N	Y	Y	N	Y	Y	Y	69
Loh et al., 2019 (USA) (3)	Y	Y	Y	N	N	Y	N	Y	Y	N	Y	Y	Y	69
McIsaac et al., 2022 (Canada) (43)	Y	Y	Y	Y	Y	Y	N	Y	Y	Y	Y	Y	Y	92
Mikkelsen et al., 2022 (Denmark) (9)	Y	Y	Y	N	Y	Y	Y	Y	Y	Y	Y	Y	Y	92
Owusu et al., 2020 (USA) (40)	Y	Y	Y	N	N	Y	N	Y	Y	N	Y	Y	Y	69
Owusu et al., 2022 (USA) (41)	Y	Y	Y	Y	Y	Y	Y	Y	Y	N	Y	Y	Y	92
Piraux et al., 2020 (Belgium) (18)	Y	Y	Y	Y	Y	Y	Y	Y	Y	N	Y	Y	Y	92
Rutkowska et al., 2021 (Poland) (44)	Y	Y	Y	Y	Y	Y	Y	Y	Y	Y	Y	Y	Y	100
Sommer et al., 2020 (Denmark) (42)	Y	N	Y	N	N	Y	N	Y	Y	N	Y	Y	N	61

Y, yes; N, no.

## 4 Discussion

This review has shown that physical exercise interventions have evidence through strategies such as individual, group, and home exercise, as well as multimodal exercise programs, through results in the physical health and quality of life of older adult cancer patients. However, the studies analyzed in this review had limitations regarding the measurement of polypharmacy and health literacy of study participants.

Studies have confirmed the benefits of physical exercise interventions for cancer patients through different methodologies (14, 45, 46). Strategies range from combined supervised resistance training to home-based exercise and recreational activities (45). Benefits have also been identified in improving the quality of life of older adult cancer patients in RCTs for pre- and post-rehabilitation programs for surgery in cancer patients (26, 47, 48).

The literature presents several evidence of the beneficial effects of physical exercise in different modalities as a complementary intervention during the cancer treatment period treatment (18, 30, 32). The most common strategies used in previous studies were high-interval training (HIIT) or resistance training (RES), which has benefits on cancer treatment-related fatigue (CTRF) and functional exercise capacity in cancer patients undergoing radiotherapy (RT) (30, 32, 49). It is important to note that studies have shown that exercise intervention had two physical benefits: aerobic capacity and range of motion of the limbs, increased physical exercise, improved health and quality of life (29, 50, 51).

Studies have compared the intervention effects of a long-term physical exercise program through continuous moderate-intensity aerobic training (MICT), RES compared to a usual care group in cancer patients, identifying similar results in the improvement of fatigue, quality of life, exercise capacity, superior muscle strength (52).

Another study using various interventions in conjunction with chemotherapy treatment for cancer patients did not show positive results from the MICT and RES interventions in terms of improving fatigue and quality of life among the participants. However, they showed more significant results than the usual care group regarding self-esteem, aerobic fitness and body fat percentage, muscle strength, and lean body mass. Courneya et al. (53), reducing fat mass, ensuring the risks of overweight and obesity as well as other comorbidities arising from this condition (23, 54, 55). It is worth noting that the issue of attendance at group activities was an essential factor in the results presented by the study participants (22, 53).

Even after the presentation of evidence of physical exercise interventions, cancer patients continue to show a reduction in the level of physical activity during treatment (56). Future studies must develop intervention programs with participants at the beginning of the treatment of cancer diseases to prevent the worsening of symptoms and guarantee the continued long-term practice of older adult cancer patients.

This issue directly impacts the rate of adherence and completion of the study and consequently the long-term benefits of physical activity. Researchers have pointed to ways of increasing the rate and continuity of patients, such as individual supervision by a professional, accessibility and location close to the place of treatment and the practice of physical exercise, and finally, the holding of physical exercise sessions near the usual treatment sessions (18).

Considering the emotional issues of cancer patients, such as limitations in self-perception of vitality, anxiety, and depression, thus being affected by the psychosocial impact of the disease, physical exercise interventions have also shown emotional benefits through previous studies (29, 57).

There are two ways of explaining the benefits of physical activity on the patient’s emotional condition and quality of life, the first being related to a decrease in chronic inflammation, thus reducing mood-related disorders. Secondly, there is an association between the brain circuits that process information about the perception of bodily sensations and mood disorders, which is improved by physical exercise (3).

Research has shown that light or moderate intensity continuous activity is sufficient to improve mood, even though it is a secondary objective in physical activity programs, since physical rehabilitation, which is the main objective, is recommended by researchers to be adapted to each participant, especially in older adult cancer patients (58). Therefore, it would be necessary for future RCT studies to identify the specific type of physical exercise, frequency, and duration suitable for each particular physical and emotional outcome (3).

Some reinforcement strategies for improving performance in patient empowerment show positive results in patients’ self-confidence, self-management, and decision-making during cancer treatment. The strategies are based on educational materials, self-management techniques, and electronic message reminders. They add value to the participants’ relationship of trust with health professionals and the intervention program, thus increasing the participants’ satisfaction with the cancer treatment process (34, 59, 60).

Another type of physical exercise intervention that showed promising results was the multimodal physical exercise program. Other RCT studies have identified significant levels of clinical improvement in cancer patients participating in a multimodal exercise intervention (9).

However, previous research still shows more significant evidence in younger and middle-aged cancer patients, with less evidence in studies of older adult cancer patients (9). A systematic review designed to analyze multimodal physical exercise interventions with older adult cancer patients identified heterogeneous studies as well as quality of life measures among the studies analyzed (61). Another systematic review study of mixed physical exercise interventions with older adult cancer patients showed benefits in physical performance, muscle strength, and physical exercise level, but the overall conclusions of the studies were limited (62).

In a broader discussion of health equity, research has identified the benefits of physical exercise programs and the impact on mortality and cancer recurrence in patients at a general level (40, 63). Although the importance of developing more studies with older adult cancer patients is highlighted, as they have a more significant functional decline and a higher risk of death (40, 64), it is worth highlighting the need to direct studies and research toward more vulnerable patients due to the lower rates of physical activity levels compared to the less vulnerable population, so promoting actions, programs, and policies to adopt healthier behaviors in this population is essential to reduce health disparities (65).

However, the studies carried out on physical exercise interventions with older adult patients with lung cancer showed greater benefits in terms of physical, psychological and wellbeing aspects compared to physical exercise interventions with patients with other types of cancer.

It should be noted that the review has some limitations, the first of which is the variety of intervention methodologies used in the studies and the respective results. The second is related to the failure to identify health literacy and polypharmacy assessment measures in the studies analyzed, where the study points to ways of reducing this gap in public health (66). The third relates to the restrictive search strategy, which may exclude potentially relevant documents. The strengths of the review were the use of a standardized protocol and evaluation instrument, as well as the inclusion of randomized clinical trial studies, increasing the possibility of the level of evidence of the studies.

## 5 Conclusion

Physical exercise interventions, such as individual, group, and home exercise, as well as multimodal exercise programs, have shown positive results. These interventions contribute to physical and mental health recovery and the quality of life of older adult cancer patients. This evidence supports researchers, public health workers, and managers in developing strategies to promote the health of older adult patients.

It is essential to encourage older people to participate in physical exercise to stay as healthy as possible. This is a difficult task, especially for those who have survived episodes such as cancer treatment, but it is no less critical. This group needs more care and attention to maintain a higher quality of life and greater wellbeing. Studies assessing how health interventions can help are welcome and increasingly necessary.

Future studies could explore physical exercise interventions with polypharmacy and health literacy measurement tools to identify specific interventions to optimize the physical and mental health care process and the quality of life of older adult cancer patients.

## Data Availability

The original contributions presented in the study are included in the article/Supplementary material, further inquiries can be directed to the corresponding author.
